# UPLC‐QTOF‐MS/MS and Antifungal Activity of a Fraction Enriched in Saponins From *Sarcomphalus joazeiro* Against *Candida* spp

**DOI:** 10.1002/bmc.70509

**Published:** 2026-06-08

**Authors:** Ana Raquel Pereira da Silva, Maria do Socorro Costa, Nara Juliana Santos Araújo, Gabriel Gonçalves Alencar, Talysson Felismino Moura, Victor Juno Alencar Fonseca, Antônia Thassya Lucas dos Santos, Maria Flaviana Bezerra Morais‐Braga, Erlânio Oliveira de Souza, Josean Fechine Tavares, Paulo Riceli Vasconcelos Ribeiro, Jacqueline Cosmo Andrade‐Pinheiro, Henrique Douglas Melo Coutinho

**Affiliations:** ^1^ Graduate Program in Biotechnology Universidade Estadual do Ceará (UECE) Fortaleza Ceará Brazil; ^2^ Laboratory Applied Microbiology (LAMAP) Universidade Federal do Cariri (UFCA) Brejo Santo Ceará Brazil; ^3^ Laboratory of Microbiology and Molecular Biology (LMBM) Universidade Regional do Cariri (URCA) Crato Ceará Brazil; ^4^ Department of Biological Chemistry Universidade Regional do Cariri (URCA) Crato Ceará Brazil; ^5^ Faculdade de Tecnologia do Cariri (FATEC) Juazeiro do Norte Ceará Brazil; ^6^ Department of Pharmaceutical Sciences Universidade Federal da Paraíba (UFPB) João Pessoa Paraíba Brazil; ^7^ Natural Products Chemistry Multiuser Laboratory (LMQPN) Embrapa Tropical Agroindustry Fortaleza Ceará Brazil

**Keywords:** antifungal activity, flavonoids, liquid chromatography, saponins

## Abstract

The development of antifungal resistance is a complex process that involves the interaction between hosts, drugs, and microbial factors, all of which contribute to therapeutic ineffectiveness. For this reason, studies on natural products as possible therapeutic alternatives are increasingly necessary in order to gain a better understanding of plant compounds that may be more effective in treating infections caused by fungal pathogens. In this context, this study aimed to investigate the antifungal potential of the saponin‐enriched fraction of the *Sarcomphalus joazeiro* species against the *Candida albicans* , *Candida tropicalis* , and *Candida krusei* strains. The selection of this species for study is due to its rich phytochemical composition and the vast traditional knowledge of its medicinal properties. The fraction obtained from the stem bark of *S. joazeiro* was analyzed by UPLC‐QTOF‐MS/MS, in which compounds such as triterpenoids, flavonoids, acids, and saponins were identified. Therefore, the results obtained in this study contribute to understanding the antifungal potential of the *S. joazeiro* species fraction against fungal infections, especially those caused by *Candida* spp.

## 1 Introduction

The development of antifungal resistance is a complex process that involves the interaction between hosts, drugs, and microbial factors, all of which contribute to therapeutic ineffectiveness. The host's immune status also plays a crucial role because the indiscriminate and unregulated use of antifungals, together with the increase in individuals with compromised immune systems, has contributed to both the emergence of opportunistic pathogens and the increase in resistance to conventional antifungals. Thus, fungi previously considered only as commensals, such as those of the *Candida* genus, are now emerging as pathogens (Lee et al. 2017).

Due to these facts, studies on natural products as possible therapeutic alternatives are necessary for a better understanding of the mechanisms and development of drugs that are more effective in treating infections caused by fungal pathogens. In this way, natural agents are being investigated as new strategies because the incorporation of plants into the formulation of remedies in the practice of traditional therapy is a fact already recognized in the pharmaceutical industry. It is therefore a fundamental practice in various applications of traditional medicinal systems (Lee et al. 2017).

*Sarcomphalus joazeiro* , belonging to the Rhamnaceae family, is an endemic plant species typical of Brazil northeastern hinterlands (Vaou et al. 2021). It can be used in human and animal food (Silva, Rocha, et al. 2022), in the manufacture of phytocosmetics (Oliveira et al. 2020) and in folk medicine (Silva, Sousa, et al. 2022). Its biological activities include antibacterial (Rego 2019), antifungal (Andrade, Silva, Freitas, et al. 2019), antiparasitic (Bezerra‐Neto 2019), antioxidante (Rocha et al. 2022), anti‐inflammatory, and gastroprotective (Brito et al. 2020). However, research into the fraction of this species and its potential antifungal activity still lacks a specific approach. Given the need to find alternatives to conventional antifungal drugs, which are often ineffective.

In this context, this study aimed to characterize the chemical composition of the fraction of the *S. joazeiro* species, as well as to evaluate its intrinsic antifungal activity and its activity in combination with fluconazole.

## 2 Materials and Methods

### 2.1 Collection of Plant Material, the Production of the Fraction Enriched in Saponins and the Identification of Compounds by UPLC‐QTOF‐MS/MS

The fraction was obtained from the stem bark of the species *S. joazeiro* Mart. The collected bark was first dried and pulverized, and then 6 g of the powder obtained was subjected to an extraction process using a Soxhlet apparatus, with the sample being subjected to two extractions, chloroform and hydroalcoholic (5 L), respectively. After the extraction cycle, the solution obtained was concentrated using an evaporator at reduced pressure and a temperature of 40°C. Thus, the saponin‐rich fraction was obtained with a yield of 1% in relation to the mass of the starting plant material (Silva et al. 2024).

The fraction was subsequently analyzed by UPLC‐QTOF‐MS/MS to identify compounds, using an Acquity UPLC system coupled with a Quadrupole/Time‐of‐Flight (UPLC‐ESI‐QTOF‐MS/MS) (Waters Corporation, Milford, USA) at the Brazilian Agricultural Research Corporation (EMBRAPA), following the methodology of Andrade, Silva, Santos, et al. (2019). The analysis was performed on a Waters Acquity UPLC BEH column (150 × 2.1 mm, 1.7 μm), with temperature set at 40°C. The gradient elution system consisted of water with 0.1% formic acid (A) and 0.1% formic acid in acetonitrile (B), with a linear gradient ranging from 2% to 95% B (0–15 min), with 0.4 mL. The negative ESI mode was collected in the range of 110–1180, and the source temperature was fixed at 120°C. The temperature and desolvation gas were 350°C and 500 L/h, respectively. The extraction cone was 0.5 V, and the capillary voltage was 2.6 kV. Additionally, leucine–enkephalin was used as a blocking mass. MSE was the acquisition mode with the instrument controlled by Masslynx 4.1 software (Waters Corporation, Milford, USA).

### 2.2 Antifungal Tests

#### 2.2.1 Fungal Strains

The fungal strains used were *Candida albicans* (INCQS 40006–ATCC 10231), *Candida tropicalis* (INCQS 40042–ATCC 13803), and *Candida krusei* (INCQS 400095–ATCC 34135), obtained from the Culture Collection of the National Institute for Quality Control in Health (INCQS), Oswaldo Cruz Foundation (FIOCRUZ), Brazil.

#### 2.2.2 Preparation of Antifungal Agent, Fraction, and Culture Media

The antifungal used was fluconazole–isofarma at a concentration of 0.2%, equivalent to 2 mg/mL. For the tests, fluconazole was diluted in sterile distilled water to a matrix concentration of 2048 μg/mL. The fraction followed the same dilution process, taking care to homogenize the solution.

All the culture media were weighed on a precision scale and prepared according to the manufacturer, diluted in distilled water, except for Sabouraud Dextrose Broth (SDB, Laboratorios cond S.A.), which was prepared in double concentration.

#### 2.2.3 Intrinsic Activity of the Antifungal Effect of Fraction and Fluconazole

For the fungal inoculum, aliquots of the microorganisms ( *C. albicans* , *C. tropicalis* , and *C. krusei* ) were collected using a platinum loop and then solubilized in saline solution until a turbidity standard equivalent to 0.5 on the McFarland scale was obtained.

The test used 96‐well flat bottom plates to carry out the broth microdilution method described by Javadpour et al. (1996) with modifications. Initially, 100 μL of SDB was distributed in each well and then a serial dilution process was carried out with 100 μL of the fraction and fluconazole (concentrations ranged from 1024 to 16 μg/mL), after which 10 μL of the fungal inoculum was added. The last wells of the plates were reserved for checking the growth of the microorganisms, as evidenced by the presence of cloudy medium on the plates. Dilution controls (using saline solution instead of inoculum) and sterility of the medium were also carried out. The plates were incubated at 37°C for 24 h. After this period, the growth of microorganisms was observed, evidenced by the presence of cloudy medium on the plates. For the quantitative tests, the plates were taken for reading in an ELISA spectrophotometer (Termoplate) at a wavelength of 630 nm (Morais‐Braga et al. 2016). The results provided the minimum inhibitory concentration (MIC) of the products tested, as well as the 50% inhibitory concentrations (IC_50_). The tests were carried out in quadruplicate.

#### 2.2.4 Determination of Minimum Fungicidal Concentration (MFC)

To determine whether the fraction (FSJ) alone and in combination was able to affect the viability of *Candida* cells, the MFC was determined. Using a pipette, 5 μL of each well of the MIC test plate (except for the sterility control and dilution of the FSJ and fluconazole) was transferred to Petri dishes containing Sabouraud Dextrose Agar (SDA) medium, distributing according to the guide card at the bottom of the plate. After 24 h of incubation, the plates were inspected for any formation of *Candida* colonies, and the concentration at which there was no growth of fungal colonies was considered the MFC of the product (Fonseca et al. 2022).

#### 2.2.5 Combined Activity of Fraction With Fluconazole

To check the effect of the fraction combined with fluconazole, the method proposed by Coutinho et al. (2008) with modifications was used, in which FSJ was tested at a sub‐inhibitory concentration (MC/8, where MC is the matrix concentration of the fraction). A serial dilution was made with 100 μL of fluconazole and the concentrations ranged from 512 to 1 μg/mL. The last wells of the plates were reserved for controlling the growth of the microorganisms, evidenced by the presence of cloudy medium on the plates. Dilution controls of the fraction in combination with fluconazole were also carried out, as well as a sterility control of the medium. The entire test was carried out in quadruplicate and the plates were incubated at 37°C for 24 h. The reading was carried out on an ELISA spectrophotometry device (Termoplate) at a wavelength of 630 nm and the results will be used to obtain a cell viability curve (Morais‐Braga et al. 2016).

### 2.3 Statistical Analysis

Statistical analysis was carried out using GraphPad Prism 9 software and the data was expressed as geometric means. Statistical significance was assessed with a two‐factor ANOVA test followed by the Bonferroni post hoc test (where *p* < 0.05 and *p* < 0.0001 are considered significant and *p* > 0.05 is not significant).

## 3 Results and Discussion

**FIGURE 1:**
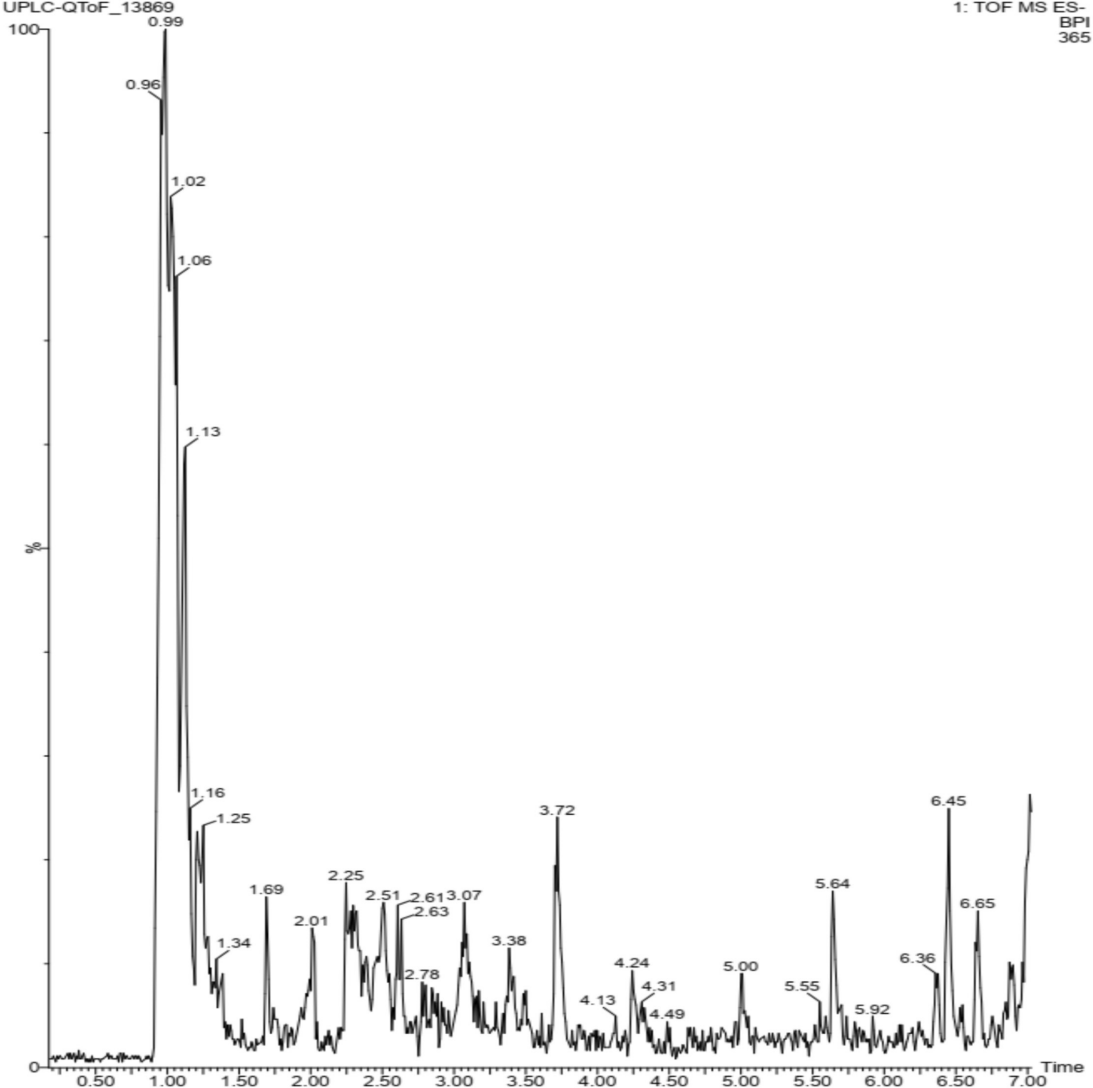
High definition mass spectrometry chromatogram (UPLC‐MS/MS) of the saponin‐enriched fraction of *Sarcomphalus joazeiro* stem barks (FSJ), in negative ionic mode.

In the studies carried out with UPLC‐ESI‐QTOF‐MS in negative mode (Figure 1), the compounds present in the *S. joazeiro* fraction were identified by high efficiency mass spectrometry, based on molecular ionic mass, retention time, fragmentation pattern and data available in the literature. Twenty compounds were identified (Silva et al. 2024). These included one nitrogen compound, two triterpenoids, three flavonoids, three saponin derivatives, five acids, and six saponins.

According to the pattern of MS fragments previously identified by Pu et al. (2017) and comparing these results with published literature. The nitrogen compound (C_33_H_27_N_2_O_9_) was identified as 5‐allyl‐1‐(2,3,4‐tris‐O‐benzoylpentofuranosyl)‐2,4(1 H,3 H)‐pyrimidinedione.

The compounds zizyphursolic acid (C_31_H_19_O_4_) and betulinic acid + OH (C_30_H_46_O_4_) (Figure 1) are two natural triterpenoids found in plant species belonging to the genus *Sarcomphalus* (Silva et al. 2024).

**FIGURE 2:**
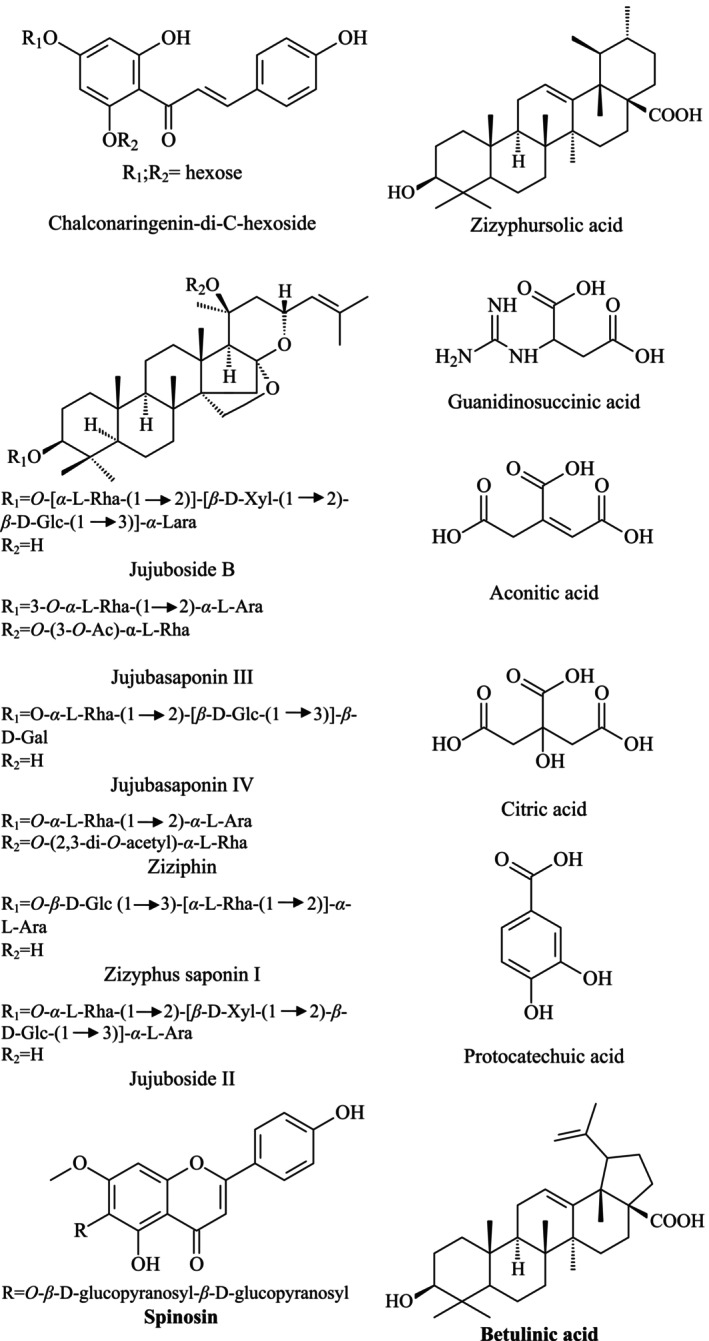
Chemical structure of the compounds identified in the fraction of *Sarcomphalus joazeiro* (FSJ).

Isospinosin (C_24_H_39_O_19_), spinosin (C_24_H_39_O_19_), and chalconaringenin‐di‐C‐hexoside (C_27_H_31_O_15_) were the compounds identified as flavonoids. The saponins identified were ziziphin (C_51_H_79_O_18_), jujuboside B (C_52_H_84_O_21_), jujuboside II (C_52_H_83_O_21_), jujubasaponin III (C_50_H_79_O_19_), jujubasaponin IV (C_49_H_79_O_20_), and zizyphus saponin I (C_47_H_75_O_17_). Additionally, the acid compounds identified were citric acid (C_6_H_7_O_7_), malic acid (C_4_H_5_O_5_), aconitic acid (C_6_H_5_O_6_), protocatechuic acid (C_7_H_5_O_4_), and guanidinosuccinic acid (C_5_H_9_N_3_O_4_) (Figure 2).

After analysis to determine the MIC of the fraction (FSJ), it was not possible to observe inhibition of fungal growth at any of the concentrations tested, indicating that the fraction did not show antifungal activity when used alone. Therefore, due to the lack of significant inhibition of the fraction throughout the concentrations evaluated, the MFC was carried out only with fluconazole, which showed fungistatic action at a concentration of 512 μg/mL.

The 50% inhibition of the microorganism population (IC_50_) for the fraction (Table 1) against *C. albicans* showed a high concentration (840.6 μg/mL) and obtained an IC_50_ of 231.2 and 212.5 μg/mL against *C. tropicalis* and *C. krusei* , respectively. The IC_50_ of the fraction against *C. krusei* was lower than that of FCZ. However, the antifungal (FCZ) showed an effect against *C. tropicalis* at a concentration of 13.23 μg/mL. When the fraction was combined with fluconazole, antagonism was observed, with an increase in concentration against *C. krusei* (318.3 μg/mL), as shown in Table 1.

**TABLE 1:** 50% inhibitory concentrations (IC_50_) of microorganisms (μg/mL) by the fraction of *Sarcomphalus joazeiro* (FSJ).

	CA INCQS 40006	CT INCQS 40042	CK INCQS 40095
Fraction	840.6	231.2	212.5
Fluconazole	717.4	13.23	294.7
FSJ + FCZ	—	—	318.3

Abbreviations: CA: *Candida albicans* ; CK: *Candida krusei* ; CT: *Candida tropicalis* ; FCZ: fluconazole; FSJ: fraction from *S. joazeiro* ; INCQS: National Institute for Quality Control in Health.

**FIGURE 3:**
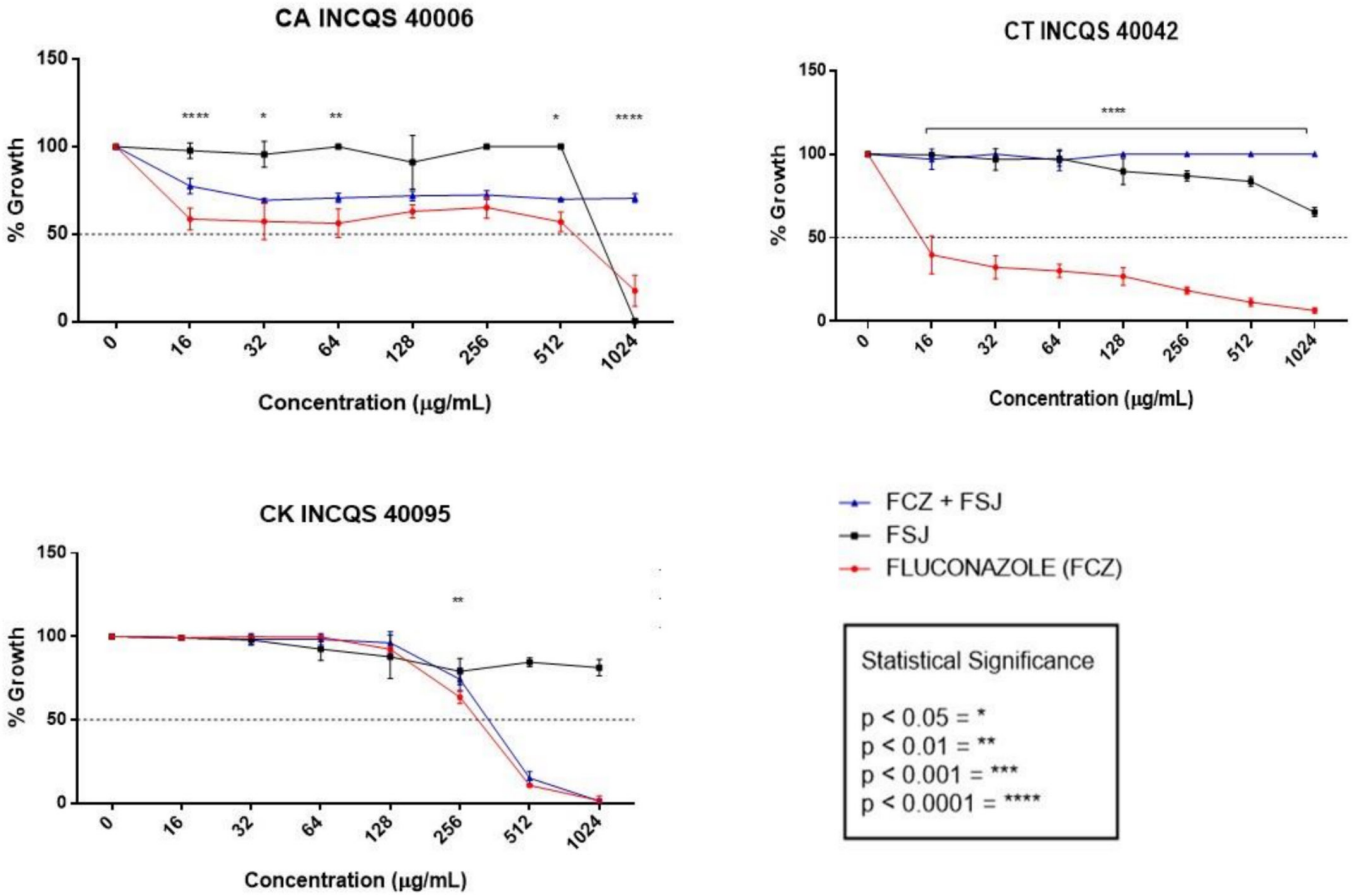
Evaluation of the modifying effect of fluconazole action by the fraction. CA: *Candida albicans* ; CT: *Candida tropicalis* ; CK: *Candida krusei* ; INCQS: National Institute for Quality Control in Health; FSJ: fraction from *Sarcomphalus joazeiro* ; FCZ: fluconazole; ns: not significant; *p < 0.05; **p < 0.01; ***p < 0.001; ****p < 0.0001—statistical significance.

Figure 3 shows the results of the cell viability of the fraction (FSJ) alone and in combination with fluconazole for the strains of *C. albicans* , *C. tropicalis* , and *C. krusei* . Therefore, when the performance of the fraction alone is observed in relation to FCZ, it can be seen that FSJ showed an almost similar performance for the *C. tropicalis* and *C. krusei* strains, in which a decrease in the percentage of microorganisms was not observed as a function of the increase in the concentration of the fraction.

It is worth noting that the FSJ in question was used at its sub‐inhibitory concentration (where the matrix concentration of the fraction was divided by 8) so that the fraction could not only show its action against the yeasts tested but also contribute at this concentration as a possible potentiator of the action of fluconazole.

In these graphs, the performance of the fraction associated with fluconazole was similar for the *C. albicans* and *C. tropicalis* strains. It was also observed that FSJ together with the antifungal FCZ showed a viability curve similar to that of the drug alone against the *C. krusei* strain (Figure 3).

The search for natural agents with bioactive properties has grown significantly, driven by the need to develop new alternatives of therapeutic importance, especially for the control of antimicrobial resistance (Lee et al. 2017). In this context, saponins, plant compounds widely distributed in nature, have aroused interest due to their diverse biological activities, including antifungal properties (Nguyen et al. 2020).

Saponins are able to form complexes with cell membrane constituents, thus altering cell permeability and leading to their destruction. According to certain studies, steroid saponins break the fungal cell membrane, making it permeable and allowing cellular components to escape. This interference can impede the vitality and growth of fungal species (Sikarwar et al. 2023).

Flavonoids exhibit diverse biological activities, including antifungal effects against species of the Candida genus. They are compounds that often inhibit fungal growth through various mechanisms, including disruption of the plasma membrane, inhibition of cell wall formation, or the efflux‐mediated pumping system (Al Aboody and Mickymaray 2020).

Secondary metabolites such as saponins, flavonoids and steroids are reported in extracts of the stem bark of *S. joazeiro* (Mauro et al. 2021). Phytochemical prospecting studies carried out on hydroalcoholic extracts of the stem bark, leaves and fruit of *S. joazeiro* have also revealed the presence of saponins, steroids and triterpenoids (Melo et al. 2012), as well as in the ethanolic extract of the leaves (Souza et al. 2023) and stem bark (Araújo et al. 2022). Chemical analysis using high performance liquid chromatography (HPLC‐DAD) identified phenolic acids in the hydroalcoholic extract of *S. joazeiro* leaves (Brito et al. 2015).

In a study carried out by Bezerra‐Neto (2019), in which the fungal susceptibility of *Candida* spp. isolates was evaluated against the enriched fraction obtained from the hydroethanolic extract of *S. joazeiro* , a significant antifungal activity was found against isolates of *C. albicans* and *Candida glabrata* , and the fraction was able to interfere with the expression of virulence factors of this group of yeasts.

Andrade et al. (2020), in a study evaluating the antifungal and modulating potential of *S. joazeiro* bark and leaf extracts, alone and in combination with fluconazole, against resistant species of the *Candida* genus, found that both extracts inhibited fungal growth, as well as showing inhibitory concentrations similar to fluconazole.

In the study carried out by Ribeiro et al. (2013), the properties of saponins extracted from the bark of *S. joazeiro* were evaluated, showing activity against *C. albicans* (156 μg/mL), using the broth microdilution method. Silva et al. (2011) and Melo et al. (2012), respectively, showed that the ethanolic and hydroalcoholic extracts of *S. joazeiro* bark were active against *C. albicans* using the agar diffusion method.

The *S. joazeiro* species also stands out for its use in folk medicine to treat infections, dandruff, or seborrheic dermatitis. According to the indications for use of the species by the population, the most commonly used parts for therapeutic, medicinal, dental, and cosmetic purposes are bark, stem, and leaves, with different forms of preparation from syrup, infusion, maceration, decoction, baths, and others (Rego 2019).

## 4 Conclusion

In conclusion, the results obtained in this study contribute to understanding the antifungal potential of the saponin enriched fraction of the *S. joazeiro* species against fungal infections, especially those caused by *Candida* spp. The data obtained from the chemical characterization of this fraction provides information on the identification of bioactive compounds present in the *S. joazeiro* species, such as triterpenoids, flavonoids, acids, and saponins. Therefore, this work highlights important scientific information for future research into new alternatives from natural products to combat microbial resistance.

## Disclosure

The authors declared that they did not use any AI tool in the text of this manuscript.

## Significance Statement

This study contributes to understanding the antifungal potential of the saponin enriched fraction of the *S. joazeiro* species against fungal infections, especially those caused by *Candida* spp. The data obtained from the chemical characterization of this fraction provide information on the identification of bioactive compounds present in the *S. joazeiro* species, such as triterpenoids, flavonoids, acids, and saponins.

## Data Availability

The data that support the findings of this study are available upon request from the corresponding author. The data are not publicly available due to privacy or ethical restrictions.
